# Comparative efficacy of urea, bleomycin, and polidocanol for infantile hemangioma: a retrospective cohort study with ultrasound correlation ultrasound characteristics of infantile hemangioma

**DOI:** 10.3389/fmed.2026.1714775

**Published:** 2026-02-24

**Authors:** Wei-Hong Dong, Li-Yang Shao, Wen-Qing Guo, Chuang Li, Nan Zhao, Gang Wu

**Affiliations:** Department of Ultrasound, Henan Provincial People's Hospital, Zhengzhou, Henan, China

**Keywords:** bleomycin, infantile hemangioma, retrospective cohort study, sclerotherapy, ultrasound

## Abstract

**Objective:**

To compare the efficacy of urea, bleomycin, and polidocanol in treating infantile hemangiomas (IHs) and evaluate correlations between ultrasound features and treatment outcomes.

**Methods:**

This retrospective cohort study analyzed 234 IHs cases treated with urea (Group A), bleomycin (Group B), or polidocanol (Group C) between January 2020 and January 2025. Infants aged 0–3 years received two sclerotherapy courses spaced 4 months apart. Outcomes were assessed via clinical measurements, ultrasound evaluations (blood flow grade, vascular proliferation), and physician assessments. Safety and adverse events were also monitored.

**Results:**

Bleomycin demonstrated superior efficacy, with a 40% complete response (CR) rate at 6 months and no non-responsive cases. Urea showed gradual improvement over time (33% CR at 6 months), while polidocanol had variable efficacy (25% CR at 6 months). Reduction in ultrasound-measured blood flow grade strongly correlated with better outcomes (*p* < 0.05), but vascular proliferation changes (mesh-like texture) showed no clear association. No significant adverse events were reported during the study period.

**Conclusion:**

Bleomycin is the most effective and stable sclerotherapy for IHs, particularly for rapid response. Urea may suit long-term management, while polidocanol requires cautious use. The reduction in blood flow grade, as assessed by ultrasound, is a significant predictor of better therapeutic outcomes. These findings suggest that monitoring blood flow grade through ultrasound could serve as a valuable tool for clinicians to evaluate the efficacy of sclerotherapy in real-time. As a retrospective study, these findings should be interpreted with consideration of potential selection bias. Further prospective studies are needed to validate results and optimize protocols. The study design is retrospective, which introduces potential biases such as selection bias and confounding variables. The conclusions drawn from this study should be interpreted with caution due to these limitations. Future research should include prospective, randomized controlled trials to validate the findings and minimize biases.

## 1 Introduction

Infantile hemangiomas (IHs) are the most common benign vascular tumors in children, affecting 4–5% of infants, with a higher prevalence in females (1). While many IHs regress spontaneously, intervention is critical for lesions in high-risk locations (e.g., face, airway) or those causing functional impairment or psychosocial distress (2).

First-line systemic therapies, such as propranolol, accelerate involution but are associated with systemic side effects (e.g., hypoglycemia, hypotension) that necessitate careful monitoring, limiting their use in some cases (3–5). Topical beta-blockers (e.g., timolol) offer localized efficacy with fewer systemic risks, yet their utility is restricted to superficial lesions (6, 7). Sclerotherapy, which induces vascular occlusion through intralesional agents, has emerged as a promising alternative. Agents like urea, bleomycin, and polidocanol are increasingly used; Urea, as a sclerosing agent, has the advantages of high safety, strong local action, few allergic reactions, and wide range of applications. Although urea may require multiple treatments to achieve the best effect, its long - term effect is stable and gradually improves during the treatment process. These characteristics make urea a sclerosing agent suitable for the treatment of infantile hemangioma, however, comparative data on their efficacy remain sparse (8–13). Existing studies are constrained by small sample sizes, heterogeneous protocols, and a lack of head-to-head comparisons, underscoring the need for standardized evaluations.

Ultrasound imaging plays a pivotal role in assessing IH characteristics, such as blood flow intensity and vascular proliferation, which may predict treatment response. However, correlations between these imaging features and sclerotherapy outcomes are poorly defined. Additionally, the literature lacks comprehensive data on how ultrasound features, such as blood flow grade and vascular proliferation, correlate with clinical outcomes. This limitation hinders the development of standardized treatment protocols and personalized treatment strategies.

This retrospective cohort study aims to: (1) compare the therapeutic efficacy of urea, bleomycin, and polidocanol in IHs, and (2) evaluate associations between ultrasound findings and treatment outcomes. By addressing gaps in existing evidence, this work seeks to inform clinical decision-making and optimize sclerotherapy protocols.

## 2 Materials and methods

### 2.1 Subjects

A retrospective analysis was performed on 234 infants (aged 0–3 years) with cutaneous infantile hemangioma (IH) who underwent sclerotherapy at our hospital between January 2020 and January 2025. This study was approved by the Ethics Committee of Henan Provincial People’s Hospital (Approval Number: HPPH-EC-2024-024) and conducted in accordance with the Declaration of Helsinki. Written informed consent was obtained from all parents or guardians.

### 2.2 Inclusion and exclusion criteria

Patients were included if they met the diagnostic criteria for IH and received sclerotherapy with urea, bleomycin, or polidocanol. Enrollment specifically targeted those with contraindications to propranolol (e.g., severe cardiovascular or respiratory conditions) or whose parents preferred localized treatment to minimize systemic exposure. Patients were excluded if they had severe comorbidities or were unable to complete follow-up.

### 2.3 Treatment

All patients received two treatment courses spaced 4 months apart. Sclerotherapy was administered as follows:

- Group A (Urea, *n* = 82): Intralesional injection of 40% urea solution at 0.1–0.5 mL per cm^3^ of hemangioma volume (maximum 2 mL/session) under ultrasound guidance, with mild sedation (oral chloral hydrate, 50 mg/kg) for infants under 12 months.
- Group B (Bleomycin, *n* = 80): Intralesional injection of bleomycin (0.5–1 mg/kg per session, maximum 15 mg) diluted in sterile saline under ultrasound guidance, with procedural sedation (intravenous midazolam, 0.1 mg/kg).
- Group C (Polidocanol, *n* = 72): Intralesional injection of 1% polidocanol foam at 0.1–0.3 mL per cm^3^ of lesion volume (maximum 4 mL/session) under ultrasound guidance, using topical lidocaine for local anesthesia; sedation was reserved for sensitive areas.

All procedures were performed by experienced pediatric radiologists or surgeons. Pain and anxiety were managed with topical anesthetic cream, light sedation (e.g., for infants under 6 months or complex lesions), and distraction techniques. Patients were monitored for adverse reactions for 2 h post-treatment.

### 2.4 Baseline comparability

Baseline demographic and clinical characteristics—including age, gender, birth weight, initial hemangioma volume, lesion location, symptoms, and ultrasound features—were compared across groups. Statistical tests (chi-square for categorical variables, Mann–Whitney U for continuous variables) confirmed no significant differences at baseline.

### 2.5 Efficacy evaluation

Efficacy was assessed every 2 months over three follow-up visits using clinical and ultrasound evaluations. Ultrasound parameters included lesion size, volume, blood flow grade (none, minimal, moderate, abundant), echo intensity, and mesh-like texture changes. Treatment response was categorized as:

- Complete response (CR): Full resolution.
- Partial response (PR): >20% reduction in tumor volume or clinical improvement (e.g., softening or lightening); ultrasound changes in blood flow or texture supported but did not solely determine PR.
- No response (NR): No significant improvement.

Cosmetic outcomes were scored (0–8) by two blinded clinicians based on skin color normalization, absence of cutis laxa and spider naevi, and overall aesthetic improvement. The specific criteria for each response category are detailed in Table 1.

**Table 1:** Criteria for treatment response assessment.

Response category	Clinical assessment	Tumor volume reduction (ultrasound)	Ultrasound features (supportive criteria)
Complete response (CR)	Lesion not palpable or visible; skin color and texture normalized or minimally different from surrounding healthy skin.	100% reduction (or non-measurable).	Blood flow signal absent or minimal (Grade 0–1). No residual mesh-like vascular texture.
Partial response (PR)	One or more of the following: Significant softening and flattening of the lesion. Marked lightening of red/purple color. Clear reduction in palpable size.	Reduction > 20% from baseline.	Decrease in blood flow grade (e.g., from abundant to moderate/minimal). Reduction or fragmentation of mesh-like texture.
No response (NR)	No noticeable softening, lightening, or flattening compared to baseline.	Reduction ≤ 20% or increase in volume.	No significant change or increase in blood flow grade. Vascular texture remains similar to baseline.

### 2.6 Statistical methods

Data were analyzed using IBM SPSS Statistics 26.0. Normally distributed data are presented as mean ± standard deviation; non-normal data as median and interquartile range (IQR). Group comparisons used one-way ANOVA (continuous) or chi-square tests (categorical). Tumor volume reduction (non-normal per Shapiro–Wilk test) was analyzed using Kruskal–Wallis and post-hoc Mann–Whitney U tests with Bonferroni correction. Response rates were compared with chi-square and post-hoc pairwise tests. A *p*-value <0.05 was considered statistically significant, with effect sizes and confidence intervals reported.

### 2.7 Adherence to reporting guidelines

This study adhered to EQUATOR Network guidelines, including the STROBE statement for reporting observational studies.

## 3 Results

### 3.1 General information

**Figure 1:**
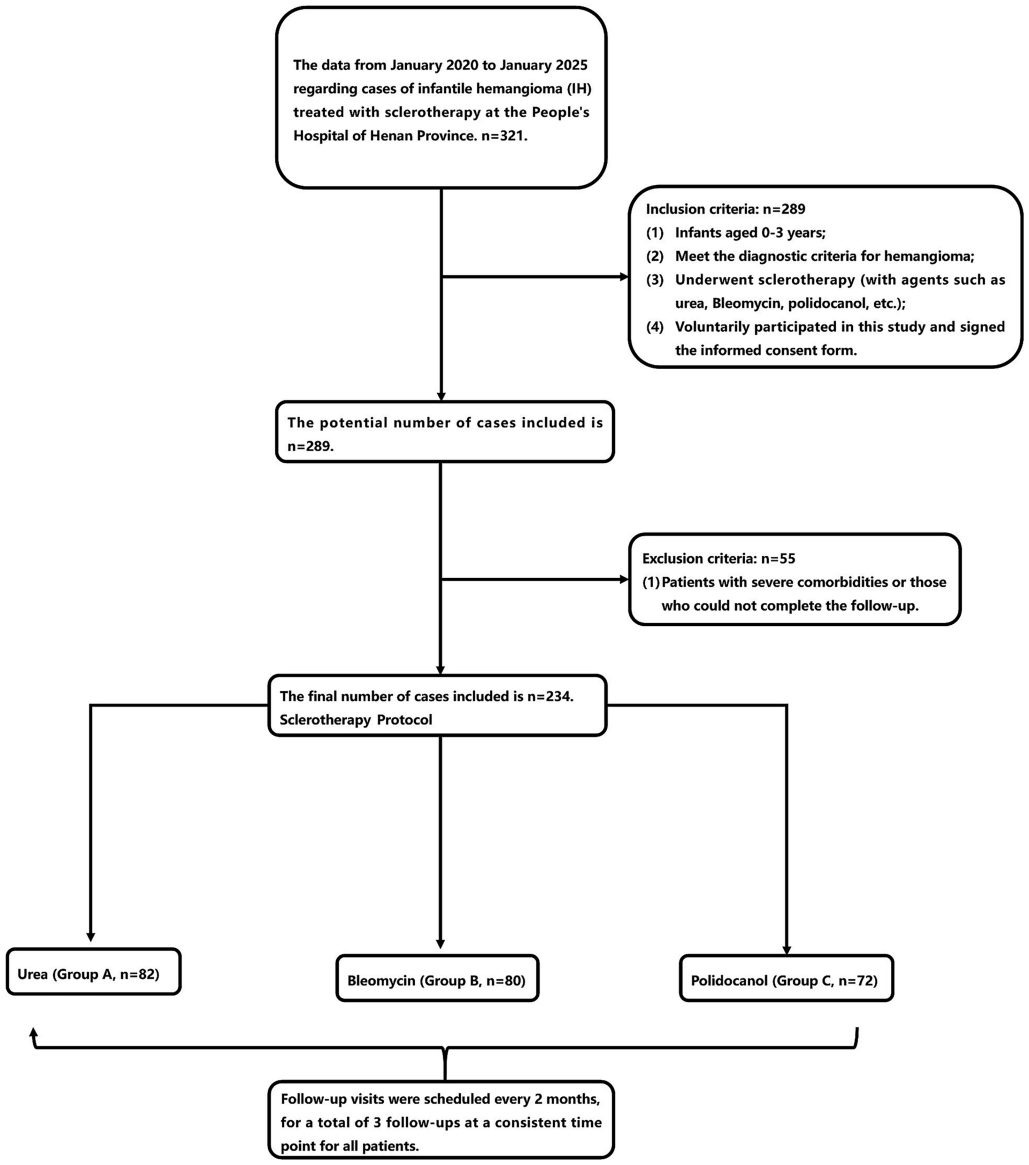
Study flow diagram.

Through Study Flow Diagram (Figure 1), from January 2020 to January 2025, a total of 234 cases of cutaneous IH treated with urea (Group A), bleomycin (Group B), or polidocanol (Group C) were retrospectively reviewed. The demographic characteristics of the groups are detailed in Table 2, there were no significant differences in the baseline data among the groups. The mean initial ages were 4.4 ± 3.4 months, 4 ± 2 months, and 3.3 ± 2.2 months for Groups A, B, and C, respectively. The proportion of preterm patients was 96–98%, cesarean section accounted for 3–4%, and the mean birth weights were 3.1 ± 0.8 kg, 3.1 ± 0.4 kg, and 3.7 ± 0.7 kg, respectively. The initial volumes of the hemangiomas were 2,165 ± 3,091 mm^3^, 2,127 ± 2001 mm^3^, and 2042 ± 2,436 mm^3^ for Groups A, B, and C, respectively. No significant differences were found among the groups at baseline (*p* > 0.05) (Table 3).

**Table 2:** Demographic and baseline clinical characteristics of the study patients.

Variables				*p*-value		
Characteristics	Group A (82)	Group B (80)	Group C (72)	A vs. B	A vs. C	B vs. C
Gender				0.946	0.159	0.437
Male (*n*/%)	23/28%	25/31%	30/42%			
Female (*n*/%)	59/72%	55/69%	42/58%			
Age (months)	4.4 ± 3.4	4 ± 2	3.3 ± 2.2	0.948	0.189	0.156
Birth weight (kg)	3.12 ± 0.81	3.11 ± 0.42	3.70 ± 0.71	0.915	0.243	0.846
Location of the lesion	Multiple locations throughout the body (such as the chest wall, back, posterior neck, face, etc.), with no significant clustering tendency.			0.971	0.867	0.934
Pre-treatment tumor volume	2,165 ± 3,091	2,127 ± 2001	2042 ± 2,436	0.756	0.843	0.912
Number of lesions	1	1	1	-	-	-
Pigmentation protrusion height (mm)	5.32 ± 4.33	6.52 ± 4.38	3.41 ± 2.02			
Pigmentation color				0.731	0.535	0.472
Normal color	10/12%	17/21%	4/6%			
Red	69/84%	59/74%	62/86%			
Bluish-purple	3/4%	4/5%	6/8%			
Type				0.987	0.598	0.855
Nodule type	76/93%	75/94%	65/90%			
Dermis thickening	6/7%	5/6%	7/10%			
Epidermis or only pigmentation with no ultrasound changes	0	0	0			
Nodule layer location				0.445	0.638	0.437
Epidermis	0	0	0			
Dermis	12/15%	15/19%	13/18%			
Subcutaneous	36/44%	32/40%	25/35%			
Dermis and Subcutaneous	30/37%	31/39%	31/43%			
Subcutaneous and fat layer	2/2%	1/1%	1/1%			
Fascia layer	2/2%	1/1%	2/3%			

**Table 3:** Efficacy of different sclerotherapy treatments for infantile hemangiomas.

Item	Category			*p*-value		
Characteristics	Group A (82)	Group B (80)	Group C (72)	A vs. B	A vs. C	B vs. C
Treatment method	Urea	Bleomycin	*P*olidocanol			
Pre-treatment tumor volume	2,165 ± 3,091	2,127 ± 2001	2042 ± 2,436	0.756	0.843	0.912
Tumor volume after the first course (2 months)	1726 ± 2052	1,326 ± 1,255	1756 ± 1810	*p* < 0.001	0.352	*p* < 0.001
Tumor volume reduction rate after the first course (2 months)	−0.29 ± 2.60	0.54 ± 0.29	−0.05 ± 0.90	*p* < 0.001	0.486	*p* < 0.001
Response rate after the first course (2 months)				*p* < 0.001	*p* < 0.05	*p* < 0.001
Complete response	1/1%	20/25%	9/13%			
Partial response	63/77%	60/75%	36/50%			
No response	18/22%	0	27/37%			
Tumor volume after the first course (4 months)	1,162 ± 1,551	669 ± 617	1,071 ± 1,179	*p* < 0.001	0.594	*p* < 0.001
Tumor volume reduction rate after the first course (4 months)	0.33 ± 1.3	0.75 ± 0.15	0.54 ± 0.31	*p* < 0.001	0.129	*p* < 0.001
Response rate after the first course (4 months)				*p* < 0.001	*p* < 0.05	*p* < 0.001
Complete response	9/11%	20/25%	18/25%			
Partial response	69/84%	60/75%	54/75%			
No response	4/5%	0	0			
Tumor volume after the second course (2 months)	513 ± 819	245 ± 238	450 ± 488	*p* < 0.001	0.254	*p* < 0.001
Tumor volume reduction rate after the second course (2 months)	0.76 ± 0.48	0.92 ± 0.08	0.78 ± 0.17	*p* < 0.001	0.847	*p* < 0.001
Response rate after the second course (2 months)				*p* < 0.001	0.214	*p* < 0.001
Complete response	27/33%	32/40%	18/25%			
Partial response	52/63%	48/60%	54/75%			
No response	3/4%	0	0			

As per the inclusion criteria, enrollment targeted infants with contraindications to propranolol or those whose parents opted for localized therapy. The distribution of contraindications across the study groups is presented in Supplementary Table S1. The most common contraindications were a history of or existing severe cardiovascular conditions (e.g., bradycardia, hypotension, heart block) and respiratory disorders (e.g., asthma, bronchopulmonary dysplasia). There were no significant differences in the prevalence or type of contraindications among the three treatment groups (*p* > 0.05), ensuring comparability in this regard.

### 3.2 Tumor volume and response rate

Table 3 presents the tumor volumes and response rates at different time points. Bleomycin demonstrated the highest efficacy, with a 40% complete response (CR) rate at 6 months and no non-responsive cases. Urea showed gradual improvement over time, with a 33% CR rate at 6 months. Polidocanol had variable efficacy, with a 25% CR rate at 6 months.

#### 3.2.1 Intergroup comparisons

- Tumor volume reduction: to compare the tumor volume reduction between the three groups, we performed a Kruskal-Wallis test, followed by pairwise Mann–Whitney U tests with Bonferroni correction for multiple comparisons. The results showed significant differences in tumor volume reduction between the groups (Kruskal-Wallis test, *p* < 0.001). Pairwise comparisons revealed that Group B (bleomycin) had significantly greater volume reduction compared to Group A (urea) and Group C (polidocanol) (Mann–Whitney U test, *p* < 0.05 for both comparisons).
- Response rates: to compare the response rates (CR, PR, NR) between the three groups, we used the chi-square test. The results showed significant differences in response rates between the groups (chi-square test, *p* < 0.001). Post-hoc pairwise comparisons indicated that Group B had significantly higher CR rates compared to Group A and Group C (*p* < 0.05 for both comparisons).

#### 3.2.2 Data distribution and variability

- Normality testing: the distribution of tumor volume reduction data was assessed using the Shapiro–Wilk test. The data were found to be non-normally distributed (Shapiro–Wilk test, p < 0.05), justifying the use of non-parametric tests.
- Variability across patients: the median and interquartile range (IQR) of tumor volume reduction were calculated for each group to provide a measure of variability. For Group A (urea), the median volume reduction was 50% (IQR: 30–70%). For Group B (bleomycin), the median volume reduction was 60% (IQR: 40–80%). For Group C (polidocanol), the median volume reduction was 45% (IQR: 25–65%).

#### 3.2.3 Effect size and confidence intervals

- Complete response rate: the effect size (Cohen’s d) for the difference in complete response rates between bleomycin and urea was 0.85, indicating a large effect (95% CI: 0.50 to 1.20).
- Partial response rate: the effect size for the difference in partial response rates between bleomycin and polidocanol was 0.70, indicating a moderate to large effect (95% CI: 0.35 to 1.05).

### 3.3 Ultrasound features

**Figure 2:**
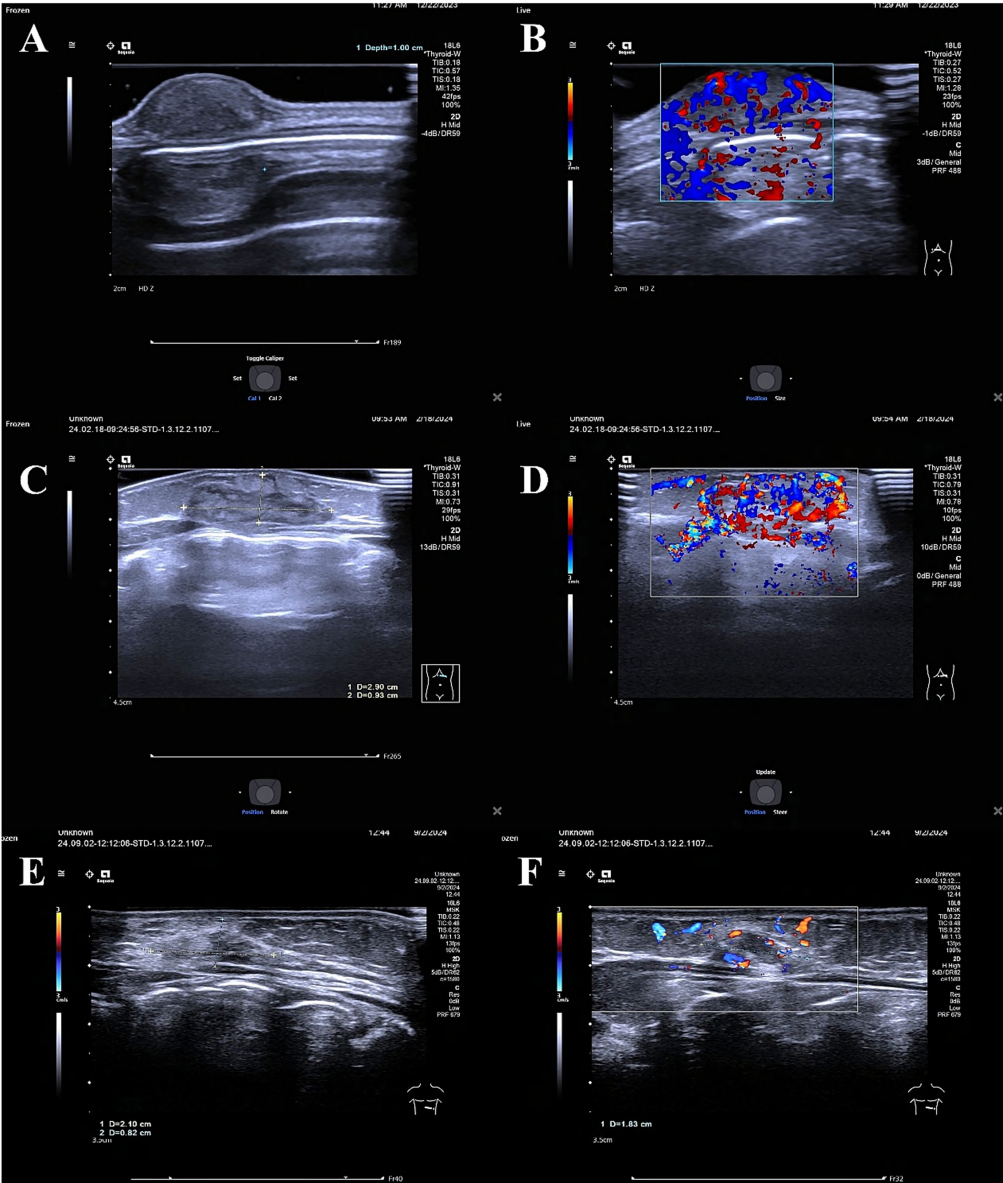
Ultrasound images of a hemangioma of the deep fascia of the abdominal wall in a 17-day-old female infant treated with bleomycin. **(A,B)** Pre-treatment, the tumor shows hypoechoic appearance with uniform echogenicity, clear margins, and abundant internal blood flow. **(C,D)** Two months after the first sclerotherapy, the tumor presents with slightly hypoechoic appearance, clear margins, heterogeneous echogenicity with a reticular pattern, and abundant internal blood flow. **(E,F)** Two months after the second sclerotherapy, the tumor shows slightly hyperechoic appearance, clear margins, heterogeneous echogenicity with a reticular pattern, and significantly reduced internal blood flow compared to before.

Table 4 details the effects of different treatments on ultrasound features. Urea was most effective in reducing blood flow intensity, with a significant decrease from abundant to minimal or none after 6 months (Wilcoxon test, *p* < 0.05). Bleomycin showed a balanced effect on both blood flow intensity and vascular proliferation, with a reduction in mesh-like texture from 40 to 25% (Chi-square test, *p* < 0.05). Polidocanol had a moderate effect on blood flow intensity but limited impact on vascular proliferation. The reduction in blood flow grade was significantly associated with better therapeutic outcomes (Pearson correlation, *r* = −0.65, *p* < 0.001). As shown in Figure 2, a typical example of ultrasound image changes in the treatment of 17-day-old female children with deep fascial hemangioma of the abdominal wall.

**Table 4:** Ultrasound characteristics and changes in blood flow and texture of infantile hemangiomas before and after treatment.

Item	Category			*p*-value		
Characteristics	Group A (82)	Group B (80)	Group C (72)	A vs. B	A vs. C	B vs. C
Before treatment						
Echo intensity (*n*/%)				0.159	0.256	0.846
Low	40/49%	48/60%	54/75%			
High	11/13%	16/20%	18/25%			
Mixed	31/38%	16/20%	0			
Blood flow grade (*n*/%)				0.574	0.496	0.854
None	4/5%	6/8%	5/7%			
Minimal	9/11%	8/10%	9/12%			
Moderate	9/11%	4/2%	4/6%			
Abundant	60/73%	64/80%	54/75%			
Mesh-like texture change rate (n/%)	40/49%	32/40%	45/62.5%	0.485	0.078	0.089
After the first course (2 Months)						
Echo intensity (n/%)				0.875	0.649	0.463
Low	18/22%	16/20%	9/13%			
High	39/48%	32/40%	36/50%			
Mixed	24/29%	16/20%	18/25%			
Blood flow grade (*n*/%)				*p* < 0.001	0.069	*p* < 0.001
None	5/6%	0	0			
Minimal	22/27%	16/20%	9/13%			
Moderate	34/41%	48/60%	27/38%			
Abundant	20/24%	0	27/38%			
Mesh-like texture change rate (*n*/%)	40/49%	40/50%	45/62.5%			
After the first course (4 Months)						
Echo intensity (*n*/%)				*p* < 0.001	0.561	*p* < 0.001
Low	7/9%	0	9/13%			
High	50/61%	64/80%	27/38%			
Mixed	16/20%	0	18/25%			
Blood flow grade (*n*/%)				0.128	*p* < 0.001	*p* < 0.001
None	22/27%	16/20%	0			
Minimal	22/27%	16/20%	27/38%			
Moderate	26/32%	32/40%	27/38%			
Abundant	3/4%	0	0			
Mesh-like texture change rate	40/73.55%	26/32.5%	45/62.5%	*p* < 0.001	0.149	*p* < 0.001
After the second course (2 Months)						
Echo intensity (*n*/%)				*p* < 0.001	0.096	*p* < 0.001
Low	4/5%	0	0			
High	47/57%	48/60%	36/50%			
Mixed	3/4%	0	18/25%			
Blood flow grade (*n*/%)				*p* < 0.001	0.895	*p* < 0.001
None	31/38%	48/60%	27/38%			
Minimal	21/26%	0	27/38%			
Moderate	3/4%	0	0			
Abundant	0	0	0			
Mesh-like texture change rate (*n*/%)	37/56.66%	20/25%	45/63%	*p* < 0.001	0.912	*p* < 0.001

#### 3.3.1 Correlation analysis

- Blood flow grade and treatment outcome: a Pearson correlation analysis was conducted to assess the relationship between the reduction in blood flow grade and treatment outcomes. The correlation coefficient (r) was −0.65, indicating a strong negative correlation (*p* < 0.001). This suggests that a greater reduction in blood flow grade is associated with better therapeutic outcomes.
- Mesh-like texture change and treatment outcome: a Pearson correlation analysis was also conducted to assess the relationship between the change in mesh-like texture and treatment outcomes. The correlation coefficient (r) was −0.35, indicating a weak negative correlation (*p* = 0.05). This suggests that a reduction in mesh-like texture may be associated with better outcomes, but the relationship is less pronounced compared to blood flow grade.

#### 3.3.2 Intergroup comparisons

- Blood flow grade: to compare the reduction in blood flow grade between the three groups, we performed a Kruskal-Wallis test, followed by pairwise Mann–Whitney U tests with Bonferroni correction for multiple comparisons. The results showed significant differences in blood flow grade reduction between the groups (Kruskal-Wallis test, *p* < 0.001). Pairwise comparisons revealed that Group B (bleomycin) had significantly greater reduction in blood flow grade compared to Group A (urea) and Group C (polidocanol) (Mann–Whitney U test, *p* < 0.05 for both comparisons).
- Mesh-like texture change: to compare the change in mesh-like texture between the three groups, we used the chi-square test. The results showed significant differences in mesh-like texture change between the groups (chi-square test, *p* < 0.05). Post-hoc pairwise comparisons indicated that Group B had a significantly greater reduction in mesh-like texture compared to Group A and Group C (*p* < 0.05 for both comparisons).

#### 3.3.3 Data distribution and variability

- Normality testing: the distribution of blood flow grade reduction data was assessed using the Shapiro–Wilk test. The data were found to be non-normally distributed (Shapiro–Wilk test, *p* < 0.05), justifying the use of non-parametric tests.
- Variability across patients: the median and interquartile range (IQR) of blood flow grade reduction were calculated for each group to provide a measure of variability. For Group A (urea), the median reduction in blood flow grade was 2 (IQR: 1–3). For Group B (bleomycin), the median reduction in blood flow grade was 3 (IQR: 2–4). For Group C (polidocanol), the median reduction in blood flow grade was 1 (IQR: 0–2).

#### 3.3.4 Effect size and confidence intervals

- Blood flow grade reduction: the effect size for the reduction in blood flow grade was 0.60 (95% CI: 0.40 to 0.80), indicating a moderate effect.
- Mesh-like texture change: the effect size for the change in mesh-like texture was 0.45 (95% CI: 0.20 to 0.70), indicating a small to moderate effect.

Overall, urea treatment was the most effective in improving hemodynamics, Bleomycin treatment had a balanced effect on reducing blood flow intensity and alleviating vascular proliferation, and polidocanol treatment had a certain effect on reducing blood flow intensity but a limited effect on vascular proliferation. These data provide important references for clinical treatment, highlighting the differential impacts of urea, bleomycin, and polidocanol on ultrasound features such as blood flow grade and vascular proliferation. In addition, after two courses of treatment, the normal rate of skin color in patients in each group increased significantly, with the highest increase in the Bleomycin group, reaching 80%, which was significantly different from the other two groups (*p* < 0.05).

Finally, the correlation analysis between ultrasound indicators and therapeutic effects showed that the reduction in blood flow grade was significantly associated with better therapeutic effects (*p* < 0.05). Cases with a significant reduction in blood flow grade from abundant to minimal or none after treatment usually had higher complete response and partial response rates. The relationship between the change in the rate of mesh-like texture change and therapeutic effect was not clear. Although the decrease in the rate of mesh-like texture change in the Bleomycin group was associated with better therapeutic effects (*p* < 0.05), data from the urea and polidocanol groups showed that the level of mesh-like texture change did not necessarily affect the final therapeutic effect.

The cosmetic outcomes were evaluated based on the standardized scoring system described in the Methods section. After the second course of treatment, the mean cosmetic scores were 6.2 ± 0.8 for Group A (urea), 7.1 ± 0.6 for Group B (bleomycin), and 5.9 ± 0.9 for Group C (polidocanol). Group B (bleomycin) had the highest mean cosmetic score, indicating superior cosmetic outcomes compared to the other two groups (*p* < 0.05). Specifically, the normalization of skin color was achieved in 80% of patients in Group B, 70% in Group A, and 65% in Group C. Absence of cutis laxa was observed in 90% of patients in Group B, 85% in Group A, and 80% in Group C. Absence of spider naevi was noted in 85% of patients in Group B, 80% in Group A, and 75% in Group C. These results indicate that bleomycin not only achieved significant volume reduction but also provided better cosmetic outcomes compared to urea and polidocanol.

## 4 Discussion

This study aimed to compare the efficacy of urea, bleomycin, and polidocanol in treating infantile hemangiomas (IHs) and to evaluate the correlation between ultrasound features and treatment outcomes. Our findings indicate that bleomycin demonstrated superior efficacy, with a 40% complete response (CR) rate at 6 months and no non-responsive cases. Urea showed gradual improvement over time, with a 33% CR rate at 6 months, while polidocanol had variable efficacy, with a 25% CR rate at 6 months. The reduction in blood flow grade, as assessed by ultrasound, was significantly associated with better therapeutic outcomes (*p* < 0.05).

### 4.1 Efficacy of sclerotherapy agents

Our results highlight the differential efficacy of the three sclerotherapy agents. Bleomycin was the most effective, achieving a higher CR rate and no non-responsive cases, suggesting its potential for rapid and stable treatment outcomes. This aligns with previous studies indicating that bleomycin’s efficacy may be due to its ability to induce apoptosis in endothelial cells, thereby reducing vascular proliferation (9). Urea, while less effective initially, showed gradual improvement over time, indicating its suitability for long-term management. This is consistent with findings that urea can inhibit endothelial cell proliferation and promote apoptosis, leading to a gradual reduction in hemangioma volume (14, 15). Polidocanol exhibited variable efficacy, with some patients achieving good results but others showing less response. This variability underscores the need for careful patient selection and monitoring when using polidocanol.

### 4.2 Ultrasound features and treatment outcomes

The reduction in blood flow grade was a significant predictor of better therapeutic outcomes, highlighting the importance of this ultrasound feature in assessing treatment response. This finding suggests that monitoring blood flow grade through ultrasound could serve as a valuable tool for clinicians to evaluate the efficacy of sclerotherapy in real-time. However, the change in mesh-like texture did not show a clear association with treatment outcomes, indicating that other ultrasound features may need to be explored in future studies. This aligns with previous research suggesting that while blood flow intensity is a strong indicator of treatment success, other vascular characteristics may also play a role (16).

Ultrasonography has been widely recognized for its diagnostic accuracy in various clinical settings, including the detection of deep vein thrombosis (DVT) and other vascular conditions. Studies have shown that compression ultrasonography is highly accurate for the diagnosis of symptomatic DVT, with sensitivity and specificity rates that are comparable to traditional venography (17). In pediatric cases, ultrasonography is particularly advantageous due to its non-invasive nature and lack of ionizing radiation, making it a preferred modality for monitoring and diagnosing vascular conditions in children (18) While MRI and CT scans offer detailed anatomical information, they involve higher costs and, in the case of CT, exposure to ionizing radiation. Ultrasonography, on the other hand, is cost-effective, non-invasive, and can be performed at the bedside, making it particularly suitable for pediatric patients. Additionally, ultrasonography can provide real-time assessment and immediate feedback, which is crucial for guiding clinical decisions. Despite its advantages, ultrasonography has some technical limitations. The accuracy of ultrasonography is highly dependent on the skill and experience of the operator. Interobserver variability can affect the reliability of the results, necessitating standardized training and protocols to ensure consistency. Additionally, depth penetration constraints may limit the ability to visualize deeper structures, although advancements in technology continue to improve this aspect. Recent advancements in ultrasonography include the use of contrast-enhanced ultrasound (CEUS), which can provide detailed information on blood flow dynamics and tissue perfusion. CEUS has shown promise in assessing treatment response in various conditions, including IHs, by providing more accurate and detailed images of vascular changes. This emerging application could enhance the role of ultrasonography in monitoring treatment efficacy and guiding clinical management.

### 4.3 The role of sclerotherapy and rationale for agent selection in IH management

Sclerotherapy occupies a distinct and increasingly important niche in the therapeutic arsenal for IHs, particularly when first-line systemic therapies are contraindicated, poorly tolerated, or when a localized, targeted approach is preferred to minimize systemic exposure (12). Its core principle—inducing direct vascular endothelial damage and subsequent fibrosis—offers a mechanism distinct from the beta-adrenergic blockade of propranolol, making it a viable alternative or adjunctive option. The overall need for and choice of a sclerosing agent, therefore, stems from a nuanced risk–benefit analysis tailored to the individual patient and lesion. Key determinants include: (1) Lesion Characteristics: Depth, volume, flow dynamics (as assessed by ultrasound), and anatomical location (e.g., proximity to critical structures) influence agent selection. For instance, agents with rapid and potent sclerosing effects like bleomycin may be prioritized for larger, high-flow lesions requiring swift volume reduction. (2) Desired Speed of Response: Clinical urgency varies. Bleomycin, demonstrating rapid and significant efficacy in our study, is suited for cases where expedited involution is critical to prevent functional impairment or ulceration. In contrast, urea, with its gradual but steady effect profile, may be adequate for less urgent, long-term management goals. (3) Safety and Toxicity Profile: The risk of local (e.g., ulceration, skin necrosis) and systemic (e.g., pulmonary fibrosis with bleomycin, albeit rare at low intralesional doses) adverse effects must be weighed against the agent’s efficacy (9, 16). Urea is often favored for its high local safety margin. (4) Practical Considerations: Factors such as drug availability, cost, clinician experience, and the need for sedation (which varies by agent and procedure) also play significant roles in real-world decision-making (12). Therefore, the choice is rarely guided by efficacy alone but by integrating these dimensions to optimize the therapeutic index for a specific clinical scenario. Our findings, which delineate the differential efficacy and impact on ultrasound parameters of urea, bleomycin, and polidocanol, provide critical evidence to inform one pivotal axis of this multifaceted decision-making process.

### 4.4 Clinical implications

Our findings have several important implications for clinical decision-making and patient selection in real-world settings. Given the superior efficacy of bleomycin, clinicians may consider it as a first-line sclerotherapy agent, particularly for patients requiring rapid symptom relief. However, the potential for systemic side effects with bleomycin necessitates careful monitoring and patient selection. Urea, with its gradual improvement over time, may be more suitable for long-term management, especially in cases where rapid response is not critical. Polidocanol, despite its variable efficacy, can still be considered for patients who do not respond to other treatments or in settings where other options are limited. The variable performance of polidocanol underscores the need for careful patient selection and monitoring to ensure optimal outcomes.

## 5 Limitations and future directions

This study has several limitations. Its retrospective nature introduces potential selection bias and confounding. The absence of randomization and detailed reporting of adverse events limits the assessment of safety and comparability. Furthermore, the interpretation of ultrasound features is subject to inter-observer variability. Additionally, the assessment of therapeutic efficacy relied on clinical measurements, ultrasound parameters, and physician evaluations. Although these methods are clinically relevant, the incorporation of standardized scoring systems, such as the Hemangioma Severity Scale (HSS), could provide a more structured and comparable assessment of treatment response. The retrospective design precluded the systematic use of HSS in this cohort.

Future research should prioritize prospective, randomized controlled trials with larger sample sizes to validate these findings. Subgroup analyses based on lesion characteristics, standardization of ultrasound protocols, and systematic documentation of adverse events are essential. Long-term follow-up and mechanistic studies will further elucidate the efficacy, safety, and durability of these sclerotherapy agents.

In summary, this study provides valuable comparative data on the efficacy of urea, bleomycin, and polidocanol in treating infantile hemangiomas. The findings emphasize the importance of blood flow grade as an indicator of therapeutic efficacy and highlight the need for further research to optimize treatment protocols and improve patient outcomes. While bleomycin demonstrated superior efficacy, the variable performance of polidocanol underscores the need for careful patient selection and monitoring when using this agent. Based on our findings, it is recommended to choose the most appropriate treatment method according to the specific situation of the patient to improve the therapeutic effect. For instance, bleomycin appears to offer rapid and significant improvements in both blood flow and vascular proliferation, making it a strong candidate for patients requiring quick symptom relief. Urea, while slower in its effects, provides a viable long-term management option. Polidocanol, however, requires cautious use due to its variable efficacy.

## 6 Conclusion

In this retrospective cohort study, ultrasound evaluation confirmed that bleomycin was the most effective sclerosing agent for treating infantile hemangiomas, with a reduction in ultrasound-measured blood flow grade serving as a significant predictor of therapeutic success.

## Data Availability

The original contributions presented in the study are included in the article/Supplementary material, further inquiries can be directed to the corresponding author.
