# Supplementary material for: Comparative efficacy of urea, bleomycin, and polidocanol for infantile hemangioma: a retrospective cohort study with ultrasound correlation ultrasound characteristics of infantile hemangioma
Source: Front Med (Lausanne). 2026 Feb 24;13:1714775. doi: 10.3389/fmed.2026.1714775 (PMC12971453; doi:10.3389/fmed.2026.1714775)
Supplement: Supplementary file 1 [file Table_1.DOCX]

**Table 1. Contraindications to First-Line Systemic Therapy (Propranolol)**

| Contraindication Category | Group A  (Urea) n=82 | Group B  (Bleomycin) n=80 | Group C  (Polidocanol) n=72 | Total  n=234 | P value* |
| --- | --- | --- | --- | --- | --- |
| **Any Contraindication, n (%)** | 82 (100%) | 80 (100%) | 72 (100%) | 234 (100%) | - |
| **Cardiovascular, n (%)** | 45 (54.9%) | 42 (52.5%) | 38 (52.8%) | 125 (53.4%) | 0.954 |
| - Bradycardia | 18 | 16 | 15 | 49 |  |
| - Hypotension | 12 | 14 | 11 | 37 |  |
| - Heart Block / Arrhythmia | 10 | 8 | 8 | 26 |  |
| - Other | 5 | 4 | 4 | 13 |  |
| **Respiratory, n (%)** | 28 (34.1%) | 30 (37.5%) | 25 (34.7%) | 83 (35.5%) | 0.901 |
| - Asthma / Reactive Airway Disease | 15 | 16 | 14 | 45 |  |
| - Bronchopulmonary Dysplasia | 8 | 9 | 7 | 24 |  |
| - Other | 5 | 5 | 4 | 14 |  |
| **Parental Preference for Localized Therapy Only, n (%)** | 9 (11.0%) | 8 (10.0%) | 9 (12.5%) | 26 (11.1%) | 0.883 |
| **Other Contraindications†, n (%)** | 5(6.1%) | 4 (5.0%) | 5 (6.9%) | 14 (6.0%) | 0.856 |

*Chi-square test for comparison among the three groups.

†Includes metabolic disorders, severe hypoglycemia risk, or known hypersensitivity to beta-blockers.
